# Dalbavancin-resistant *Staphylococcus epidermidis in vivo* selection following a prosthetic joint infection: phenotypic and genomic characterization

**DOI:** 10.1093/jacamr/dlae163

**Published:** 2024-10-18

**Authors:** L Ruffier d’Epenoux, P Barbier, E Fayoux, A Guillouzouic, R Lecomte, C Deschanvres, C Nich, P Bémer, M Grégoire, S Corvec

**Affiliations:** Institut de Biologie des Hôpitaux de Nantes, Service de Bactériologie et des Contrôles Microbiologiques, CHU de Nantes, 9 quai Moncousu, 44093 Nantes Cedex 01, France; INSERM, Immunology and New Concepts in ImmunoTherapy, INCIT, UMR 1302, Nantes Université, Nantes, France; Membre du CRIOGO (Centre de Référence des Infections Ostéo-articulaires du Grand Ouest), Nantes, France; Institut de Biologie des Hôpitaux de Nantes, Service de Bactériologie et des Contrôles Microbiologiques, CHU de Nantes, 9 quai Moncousu, 44093 Nantes Cedex 01, France; Institut de Biologie des Hôpitaux de Nantes, Service de Bactériologie et des Contrôles Microbiologiques, CHU de Nantes, 9 quai Moncousu, 44093 Nantes Cedex 01, France; Institut de Biologie des Hôpitaux de Nantes, Service de Bactériologie et des Contrôles Microbiologiques, CHU de Nantes, 9 quai Moncousu, 44093 Nantes Cedex 01, France; Membre du CRIOGO (Centre de Référence des Infections Ostéo-articulaires du Grand Ouest), Nantes, France; Service des Maladies Infectieuses, Hôtel-Dieu, Centre Hospitalier Universitaire, Nantes, France; Centre d’Investigation Clinique Unité d’Investigation Clinique, Centre Hospitalier Universitaire, Nantes, France; Membre du CRIOGO (Centre de Référence des Infections Ostéo-articulaires du Grand Ouest), Nantes, France; Service des Maladies Infectieuses, Hôtel-Dieu, Centre Hospitalier Universitaire, Nantes, France; Centre d’Investigation Clinique Unité d’Investigation Clinique, Centre Hospitalier Universitaire, Nantes, France; Membre du CRIOGO (Centre de Référence des Infections Ostéo-articulaires du Grand Ouest), Nantes, France; Nantes Université, CHU Nantes, Clinique Chirurgicale Orthopédique et Traumatologique, F-44000 Nantes, France; Nantes Université, INSERM, UMRS 1229, Regeneration Medicine and Skeleton (RMeS), ONIRIS, F-44042 Nantes, France; Institut de Biologie des Hôpitaux de Nantes, Service de Bactériologie et des Contrôles Microbiologiques, CHU de Nantes, 9 quai Moncousu, 44093 Nantes Cedex 01, France; Membre du CRIOGO (Centre de Référence des Infections Ostéo-articulaires du Grand Ouest), Nantes, France; Service de Pharmacologie, CHU Nantes, Nantes, France; UMR Inserm 1235, The Enteric Nervous System in Gut and Brain Disorders, Nantes Université, Nantes, France; Institut de Biologie des Hôpitaux de Nantes, Service de Bactériologie et des Contrôles Microbiologiques, CHU de Nantes, 9 quai Moncousu, 44093 Nantes Cedex 01, France; INSERM, Immunology and New Concepts in ImmunoTherapy, INCIT, UMR 1302, Nantes Université, Nantes, France; Membre du CRIOGO (Centre de Référence des Infections Ostéo-articulaires du Grand Ouest), Nantes, France; ESGIAI (ESCMID Study Group for Implant-Associated Infections) Member

## Abstract

**Background:**

Dalbavancin is a lipoglycopeptide antibiotic with a wide spectrum of activity against Gram-positive bacteria, including MDR isolates. Its pharmacokinetic properties and administration patterns could be useful for the treatment of bone and joint infections, especially prosthetic joint infections (PJIs).

**Introduction:**

We report the case of an 80-year-old man who experienced an acute periprosthetic joint infection of his right total knee arthroplasty (TKA). A DAIR procedure was done with tissue sampling, which allowed identification of a linezolid-resistant MDR *S. epidermidis* (LR-MDRSE) strain. The patient was then treated with dalbavancin (four injections).

**Methods:**

We studied the phenotypic and genomic evolution of the strains and plasma through concentrations of dalbavancin at different points in time.

**Results:**

After four injections (1500 mg IV) of dalbavancin over a 6 month period, the dalbavancin MIC increased 4-fold. Calculated *f*AUC_0–24_/MIC ratios were 945, 1239 and 766.5, respectively, at Days 49, 71 and 106, assuming an MIC of 0.032 mg/L. The PFGE dendrogram revealed 97% similarity among all the isolates. These results suggest acquisition by the *S. epidermidis* strain of dalbavancin resistance when the patient underwent dalbavancin treatment. A 4-amino-acid deletion in the *walK* gene coinciding with the emergence of phenotypic resistance was revealed by WGS without any other relevant indels.

**Conclusions:**

Despite dalbavancin treatment with pharmacokinetic management, emerging dalbavancin resistance in *S. epidermidis* was observed, resulting in treatment failure. This outcome led to a prosthesis revision and long-term suppressive antibiotic therapy, with no recurrence of PJI after an 18 month follow-up.

## Introduction

Dalbavancin is a long-acting lipoglycopeptide active against Gram-positive bacteria, including MRSA and VRE harbouring *vanB* genes.^1–3^ Regardless of authorization for the treatment of acute bacterial skin and skin structure infections (ABSSSIs), dalbavancin is mainly used in off-label indications such as bone and joint infections (BJIs), including periprosthetic infections (PJIs), endocarditis or vascular graft infections.^4^

Its pharmacokinetic profile with a long half-life makes it particularly suitable for the treatment of BJI. In addition, dalbavancin allows spaced administrations along with good bone penetration^5^ and an excellent tolerability profile.^6^ Moreover, *in vitro* models have shown dalbavancin is also active against staphylococcal biofilm in association with rifampicin.^7–9^

Dalbavancin has a similar mechanism of action and spectrum as vancomycin but with increased potency, higher protein binding and an elimination half-life up to 60 times longer.^10^ Due to their pharmacodynamical similarities, cross-resistance among vancomycin, daptomycin and dalbavancin was observed *in vitro* after exposure to dalbavancin alone had selected for daptomycin- and vancomycin-non-susceptible strains of *Staphylococcus aureus.*^11,12^ Based on these results, the authors suggested that dalbavancin exposure may also increase the collateral risk of vancomycin and daptomycin resistance. Neutropenic murine thigh and lung infection models have shown that the 24 h drug-free period under the concentration-versus-time curve/MIC ratio (*f*AUC_0–24_/MIC) best correlated with treatment efficacy and supports the use of high doses spaced as far apart as possible.^13,14^

Dalbavancin primary resistance remains infrequent; the emergence of reduced susceptibility during dalbavancin exposure was described *in vitro* for *S. aureus.*^15^ Recently, Zhang *et al.*^16^ reported the occurrence of dalbavancin resistance associated with *walK* and *scrA* mutations during dalbavancin treatment. These mutations induced a reduction in long-chain lipid content causing a decrease in membrane fluidity.

Methicillin resistance in CoNS is frequently associated with resistance to other antibiotics, except for glycopeptides, which for many years were the drugs of choice in the treatment of staphylococcal infections. Over recent decades, treatment options for Gram-positive infections have expanded significantly with new glycopeptides, β-lactams, lipopeptides, glycylcyclines and oxazolidinones (linezolid and tedizolid). However, high rates of oxazolidinone consumption or the use of long courses of therapy promote resistance.^17^ Côrtes *et al.* and Coustillères *et al.* described MDR *Staphylococcus epidermidis* (MDRSE) ST2 clones known to be involved in hospital-acquired infections and capable of persisting and spreading for several years in the same hospital.^18,19^ These methicillin-resistant *S. epidermidis* (MRSE) clones belonged to ST2 and harboured both *cfr* and mutation in genes encoding 23S rRNA and substitution in ribosomal protein L3. This combination conferred high-level resistance to linezolid, but also to tedizolid, further limiting antimicrobial treatment options.

Here we report a case of linezolid-resistant MDRSE (LR-MDRSE) PJI with the selection of a dalbavancin-resistant clone with a resistance mechanism, albeit not yet described. We investigated the phenotypic resistance evolution of the different isolates. A comparative genomic analysis by WGS was conducted to investigate the genomic alterations that could be responsible for reduced susceptibility to dalbavancin.

## Materials and methods

### Case presentation

An 80-year-old man underwent a revision of a medial unicompartmental knee arthroplasty to total knee arthroplasty (TKA) for progressive arthritis of the lateral joint. Three weeks after surgery, the patient had a scar disunion, with large exposure of the prosthesis, suggesting an early surgical site infection.

It was subsequently decided to perform debridement, antibiotics and implant retention (DAIR) with a change of modular implants. Three bacteriological samples were collected, ultimately revealing a positive LR-MDRSE strain resistant to methicillin, rifampicin and linezolid but still susceptible to dalbavancin and ceftaroline with MICs of 0.032 and 1 mg/L, respectively [STA1, 24 days post arthroplasty (Day 24)]. A treatment shift to dalbavancin 1500 mg IV on Days 35, 49, 71 and 106, combined with doxycycline 200 mg/day for 3 months was initiated.

Seven months after the surgery, the patient was seen for his follow-up consultation. The evolution was considered unfavourable due to a loco-regional inflammatory syndrome with persistent chronic pain. A joint aspirate revealed a dalbavancin-resistant LR-MDRSE strain that was still susceptible to ceftaroline (STA3, Day 240, dalbavancin and ceftaroline MICs of 0.5/0.75 mg/L, respectively).

A revision of the knee was decided upon, with five bacteriological samples collected. As previously, they tested positive with the same LR-MDRSE strain (Day 371). Ceftaroline (1800 mg/day) and doxycycline (200 mg/day) combination treatment was administered for the next 12 weeks. The treatment turned out to be well tolerated and the evolution was favourable (with a clean, closed and non-inflammatory scar without discharge). Eighteen months after the revision TKA, the patient did not report any pain and was in good overall condition. His C-reactive protein level was <4 mg/L (reference range: <4 mg/L). However, suppressive doxycycline therapy was maintained (200 mg/day).

### Bacterial isolates

Isolates were identified by MALDI-TOF mass spectrometry (VITEK MS, bioMérieux, Marcy-l’Étoile, France). Antibiotic susceptibility testing was performed using the AST-P668 bioMérieux card using a VITEK XL automated system (bioMérieux). Dalbavancin, ceftaroline, delafloxacin, tigecycline and daptomycin MICs were determined using a 0.5 McFarland bacterial inoculum on Mueller–Hinton agar plates with gradient strips (Liofilchem, Roseto degli Abruzzi, Italy) incubated for 24 h at 35°C. Interpretation of the susceptibility was made according to the EUCAST 2022 criteria.

### PFGE

The PFGE procedure was performed as previously described.^20^ Briefly, a washed bacterial suspension was mixed with 2% SeaPlaque agarose (FMC, Rockland, ME, USA) at 55°C and allowed to solidify into plug moulds at room temperature. Chromosomal DNA was prepared in several incubation and washing steps by using EC buffer (Tris-HCl, NaCl, EDTA, deoxycholate, Sarkosyl), lysostaphin and proteinase K. After digestion of the genomic DNA by SmaI, the restriction fragments were separated by PFGE using a temperature-controlled CHEF DRIII system (Bio-Rad, Hertfordshire, UK). After staining with 0.5 μg/mL ethidium bromide, the fragments were visualized by using a UV transilluminator and then documented using a video gel documentation system. For PFGE pattern analysis, GelCompar software (Applied Maths) was applied. The dendrograms were calculated, and isolates were considered, respectively, identical or closely related when there were either 0 or ≤2 banding differences.

### WGS and genomic analysis

Briefly, WGS was performed on the four isolates using the MiSeq platform. The raw reads were trimmed, then assembled into contigs and scaffolded using, respectively, Trim Reads 2.5 and De Novo Assembly 1.5 tools from the CLC Genomic Workbench version 22.0 (QIAGEN). Contigs with length below 500 bp were discarded. Genome assemblies were submitted to PlasmidFinder for plasmid contigs identification. This prediction was expanded using an extra step. Briefly, trimmed reads were assembled by plasmidSPAdes to distinguish plasmids and chromosomes by coverage. Plasmids contigs were then mapped back to the CLC assemblies for identification using BLAST and pulled apart. The resulting four strain sets of chromosome contigs were ordered by alignment against the genome of the *S. epidermidis* RP62A reference strain (GenBank accession NC_002976.3) using the Whole Genome Alignment tool from the CLC Genomic Workbench version 22.0. Annotation of protein coding genes within ordered chromosome contigs and plasmid contigs were performed using the MicroScope platform. Prediction of resistomes were performed using the Comprehensive Antibiotic Resistance Database (CARD, version 3.0.2).

### Dalbavancin therapeutic drug monitoring

Because of the long-term duration, dalbavancin therapeutic drug monitoring was performed. Plasma trough concentrations were measured using a validated LC coupled with MS method (Acquity UPLC^®^ BEH C18 and Acquity QDA, Waters, France). The lower limit of quantification for dalbavancin was 1 mg/L and the limit of linearity was 200 mg/L. Knowing that dalbavancin is 93% bound to plasma proteins and taking into account the long terminal half-life, some authors consider a single plasma concentration measurement, at least 1 week after the injection, enough to extrapolate *f*AUC_0–24_.^21,22^ We therefore used this methodology for *f*AUC_0–24_ determination.

## Results

### Antibiotic susceptibility testing

**Figure 1.**
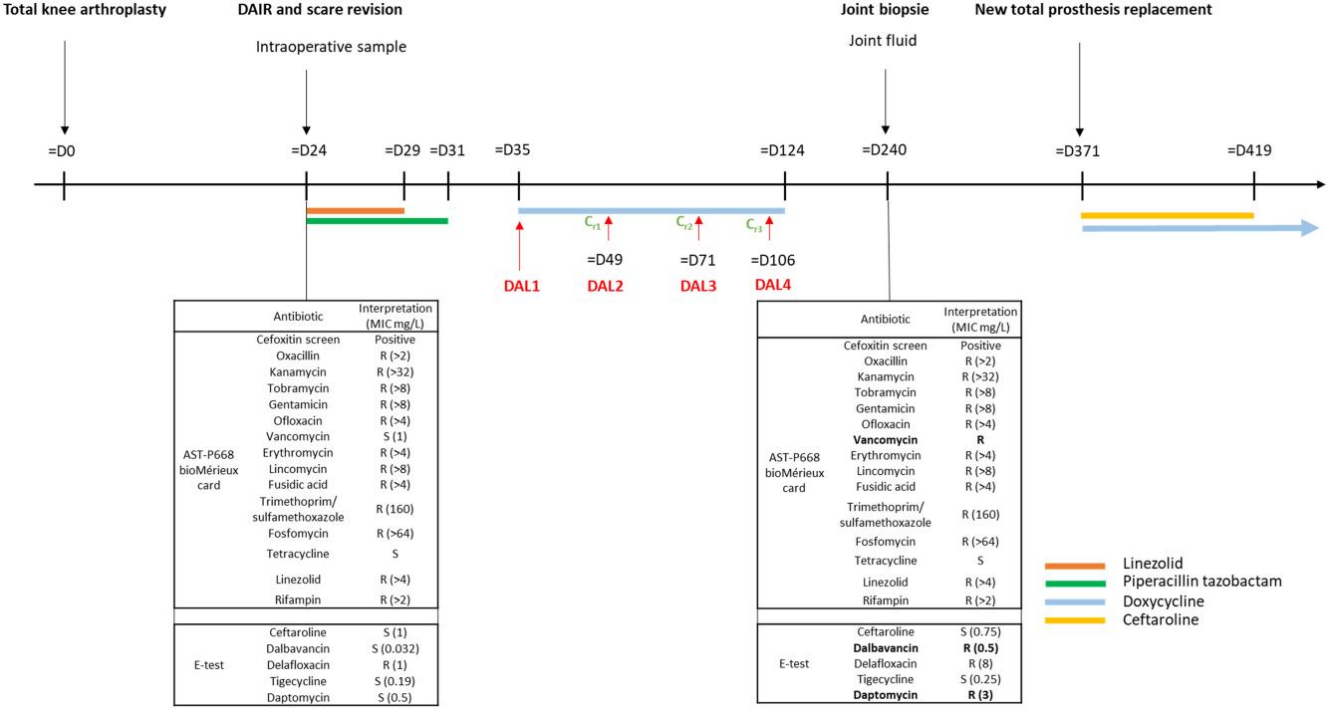
Timeline of diagnostic and therapeutic management. D, Day; DAL, dalbavancin; C_r_, residual concentration (mg/L); R, resistant; S, susceptible.

Susceptibilities to 21 antibiotics for two types of isolates (STA1 at Day24 and STA3 at Day240) are listed in Figure 1. Dalbavancin MIC increased 4-fold between the two instances (isolate 1 MIC = 0.032 mg/L; isolate 3 MIC = 0.5). It is interesting to note that, beside the increase in dalbavancin MIC, we also observed an increase of the MICs of vancomycin (>5-fold) and daptomycin (6-fold). Finally, ceftaroline, delafloxacin, tigecycline MICs were unchanged.

### Dalbavancin therapeutic drug monitoring

Three dalbavancin plasma trough concentrations (before administration) were measured. Concentrations were 18 mg/L at Day 49 (measured 14 days after the last dose), 23.6 mg/L at Day 71 (measured 22 days after the last dose) and 14.6 mg/L at Day 106 (measured 35 days after the last dose). Calculated *f*AUC_0–24_/MIC ratios were 945, 1239 and 766.5, respectively, at Days 49, 71 and 106, assuming an MIC of 0.032 mg/L. It is interesting to note that the calculated *f*AUC_0–24_/MIC ratios would be 60, 79 and 49, respectively, at Days 49, 71 and 106 assuming an MIC of 0.5 mg/L.

### PFGE

PFGE was performed after restriction by the SmaI enzyme. The macrorestriction profiles of the isolates were perfectly identical for three of them [100% similarity; two strains from episode 1 (STA1 and STA2) and one strain from episode 2 (STA4)], suggesting a strong relatedness between these isolates according to Tenover’s criteria.^23^ The last isolate [second strain from episode 2 (STA3)] had only one band difference and thus had a very similar macrorestriction profile (97% similarity), suggesting tight ties with the clone. Thus, despite the evolution towards resistance, it turns out to be clearly the same strain present in both episode 1 and episode 2, despite happening 216 days apart.

### WGS and genomic analysis

Genomic comparison analysis of the four isolates (STA1 to STA4) revealed that they all belonged to ST2 with several indels. The genomes were identical between STA1 and STA2 (strains from episode 1) and STA3 and STA4 (strains from episode 2), except for a loss of about 50 genes (mainly plasmid genes, which were not found in STA3 and STA4) and a ‘gain’ of 8 genes (mainly genes encoding ‘proteins of unknown function’ and a *merT* gene corresponding to a mercury transporter or a close homologue). All strains contained *blaZ*, *mecA*, *fusB*, *dfrC*, *norA*, *aph (2″)-Ia*, *ant (4′)-Ib*, *qacA* and *mupA* genes.

**Figure 2.**
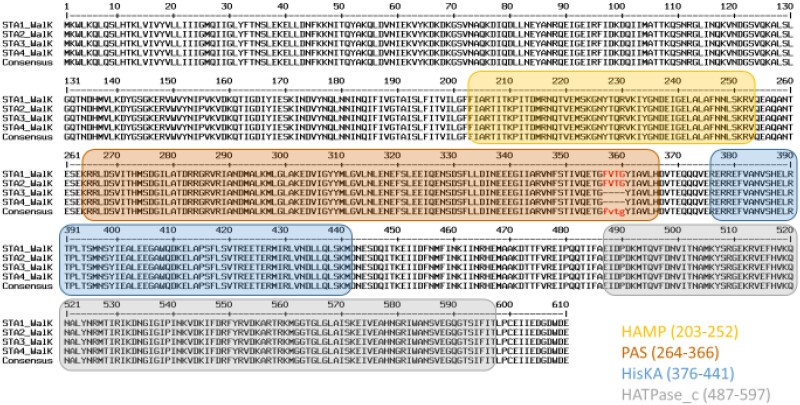
Amino acid sequences of *walK* gene alignment of the four *S. epidermidis* study isolates against NCBI Reference Sequence WP_002437327.1. Cytoplasmic domains are highlighted in colours according to InterPro analysis and classification. HAMP (InterPro entry: IPR003660): histidine kinases, adenylyl cyclases, methyl binding proteins, phosphatases domain; PAS (IPR000014): signal sensor domain; his_kinase_dom (IPR005467): histidine kinase domain. Amino acids highlighted in red were found to be deleted within the genomic sequences of STA3 and STA4 strains. STA1 and STA2 correspond to episode 1 (24 days after arthroplasty) and STA3 and STA4 correspond to episode 2 (240 days after arthroplasty).

Moreover, it is worth noting that a 12 bp deletion in the *walK* gene, was observed in STA3 and STA4 but not in STA1 and STA2 (Figure 2). Mutations in this gene have been previously described and linked to reduced dalbavancin susceptibility.^15^

### Discussion

Infections caused by ST2 *S. epidermidis* are a major problem due to their increasing resistance to antibiotics such as rifampicin and linezolid, which drastically limits antimicrobial treatment options.

Moreover, the clinical infections caused by biofilms poses a serious threat to public health. *Staphylococcus* spp. biofilms stand out as one of the most prevalent, especially in osteoarticular infection and PJI. The biofilm lifecycle represents a significant hurdle for treatment because bacterial cells become highly tolerant to a wide range of antimicrobial compounds, which are usually effective against the planktonic forms. Thus, new therapeutic strategies targeting biofilms and MDR bacteria are urgently needed. Recent studies have shown dalbavancin could successfully reduce MRSA, methicillin-resistant *S. epidermidis* and vancomycin-susceptible enterococci in biofilms.^7–9,24^ The particular characteristics of this antibiotic are its prolonged half-life (about 14 days), its good bone penetration and excellent tolerability profile.^5,6^ Thus, dalbavancin is a promising option for the treatment of infections associated with biofilms.

Here, we present an LR-MDRSE PJI case with emerging dalbavancin resistance under treatment, resulting in treatment failure and, consequently, the need for long-term suppressive antibiotic therapy.

*f*AUC_0–24_/MIC best correlates with *in vivo* efficacy of dalbavancin.^13^ Indeed, Lepak *et al.*^14^ showed that for seven isolates of *S. aureus* (including four vancomycin-intermediate *S. aureus* strains) that *f*AUC_0–24_/MIC values were, respectively, 27.1, 53.3 and 111.1 for net stasis, 1-log kill and 2-log kill. For our patient, calculated *f*AUC_0–24_/MIC ratios were 945, 1239 and 766.5, respectively, at Days 49, 71 and 106, assuming an MIC of 0.032 mg/L, but only 60, 79 and 49, respectively, assuming an MIC of 0.5 mg/L. Indeed, it is possible that between the first MIC measurement (Day 24) and the days of dalbavancin injections (Days 35, 49, 71 and 106), the MIC could have increased due to insufficient antibiotic pressure within the biofilm.

Analyses of the genomic data for the patient isolates revealed disappearance of the *walK* gene. The WalKR system, a two-component system (TCS) specific to low G + C Gram-positive bacteria, is the only essential TCS in *S. aureus* viability.^20,21^ The WalKR plays a role in cell wall biosynthesis, virulence and antibiotic resistance. Thus, the disappearance of the *walK* gene observed in the MDR strains could lead to an increase in wall thickness, explaining the observed resistance of this bacterium to dalbavancin, daptomycin and vancomycin.

Previous *in vitro* studies of *S. aureus* have demonstrated cross-resistance among dalbavancin, vancomycin and daptomycin.^11,12^ Thus, we included determination of daptomycin and vancomycin MICs for the four *S. epidermidis* isolates studied in this work. We observed a significant change in MICs of these different molecules, with an increase of 6-fold and >5 fold, respectively, for daptomycin and vancomycin, validating the cross-resistance.

Using WGS, we were able to establish that this *S. epidermidis* strain belongs to ST2. Recent studies have shown that this ST2 lineage is particularly enriched in *S. epidermidis* strains isolated from patients with PJI.^18,19^ In addition, Trobos *et al.*^25^ have shown that this ST2 lineage is responsible for the majority of relapses and is associated with multidrug resistance and high biofilm production, which may also explain the treatment failure.

For these particular strains, source control (especially bacterial inoculum) combined with adequate antibiotic concentrations is important to prevent reduced susceptibility from emerging during dalbavancin treatment.

This case highlighted that good pharmacokinetic follow-up should probably be established to monitor prolonged treatment during PJI. Antibiotic bi-monthly monitoring ought to be mandatory in order to properly manage this off-label cure.

This case raises some questions about the management of these ST2 *S. epidermidis* LR-MDRSE infections. Given the WalKR deletions, the thickening of the wall, and the capacity to overproduce biofilm, should we continue to use lipoglycopeptides on these LR-MDRSE strains or should we rather prescribe a β-lactam such as ceftaroline, which is still very active, even if the diffusion is not as good?

For these cases where strains overproduce biofilm and where surgery is not optimal (DAIR), should we continue to use dalbavancin-based monotherapy for the treatment of BJI or should we always use it in combination with rifampicin?

In our opinion, several factors contributed to this patient’s treatment failure: unsuitable surgery (DAIR); a particular bacterial strain (LR-MDRSE) that was multi-resistant and overproduced biofilm; and the lack of combination of dalbavancin with rifampicin.

Finally, further clinical research studies should be performed to optimize this treatment, especially for LR-MDRSE strains, which may have derepressed metabolic pathways leading to biofilm overproduction.
